# Consumption of a *Leuconostoc holzapfelii-*enriched synbiotic beverage alters the composition of the microbiota and microbial extracellular vesicles

**DOI:** 10.1038/s12276-019-0288-1

**Published:** 2019-08-01

**Authors:** Jinho Yang, Andrea McDowell, Eun Kyoung Kim, Hochan Seo, Kyujin Yum, Won Hee Lee, Young-Koo Jee, Yoon-Keun Kim

**Affiliations:** 1 MD Healthcare Inc., Seoul, Republic of Korea; 2 Department of Health and Safety Convergence Science, Korea University, Seoul, Republic of Korea; 3 Coenbio Co., LTD, Seoul, Republic of Korea; 4 Department of Internal Medicine, Dankook University College of Medicine, Cheonan, Republic of Korea

**Keywords:** Biological therapy, Predictive markers

## Abstract

Synbiotics, the combination of probiotics and prebiotics, are known to confer health benefits via intestinal microbiota modulation. However, significant intestinal microbiota alterations can be difficult to determine in intervention studies based on solely bacterial stool metagenomic analysis. Intestinal microbiota constituents secrete 20–200-nm-sized extracellular vesicles (EVs) containing microbial DNA, proteins, and lipids that are distributed throughout the body, providing an alternative target for microbiota metagenomic analysis. Here, we determined the impact of a synbiotic beverage enriched with the kimchi-derived bacterium *Leuconostoc holzapfelii* (*L*. *holzapfelii*) on the intestinal microbiota and local and circulatory microbiota-derived EV composition of healthy Korean adults. We isolated microbial DNA from stool bacteria, stool EVs, and urinary EVs and conducted next-generation sequencing of the 16S rDNA V3–V4 regions before and after synbiotic consumption. The species diversity of circulating urinary EVs was significantly increased after synbiotic consumption, while stool bacterial and EV diversity remained unchanged. Furthermore, we found that while a single genus was decreased among the stool bacteria constituents, stool EVs and urinary EVs showed significant alterations in four and eight genera, respectively. Blood chemistry assays revealed that synbiotic consumption significantly lowered aspartate aminotransferase (AST) serum levels, particularly in subjects with starting levels above the normal range (>40 UI/L). In conclusion, the *L*. *holzapfelii-*enriched synbiotic beverage greatly altered serum AST levels and microbial EV composition in urine and stool, while only minor changes were observed in the gut microbiota composition. Based on these findings, we suggest the potential use of microbiota-derived EVs as surrogate markers in future predictive diagnosis studies.

## Gut microbes: Vesicles reveal variations in populations

Analyzing the contents of small membrane-bound sacs called extracellular vesicles (EVs) that are released by microbes in the gut, and which can readily be sampled in stools and urine, offers an effective way to monitor gut microbial populations. Bacteria known as probiotics and chemicals that sustain them called prebiotics are commonly used to encourage health-promoting changes in gut microbe content. Detecting any changes by analyzing the bacteria in stools, however, has proved difficult. Researchers in South Korea led by Yoon-Keun Kim of MD Healthcare Inc., Seoul, and Young-Koo Jee at Dankook University, Cheonan, found that analyzing microbial EVs in stools or urine detected changes more effectively. They analyzed EVs after administering a synbiotic drink – a mixture of probiotics and prebiotics. EV analysis could assist diagnosis of health problems associated with changes in gut microbial populations.

## Introduction

The gut microbiota is a community of trillions of bacteria residing in the intestinal tract, which is comprised of ~2000 species that contribute over two million genes to the host^1,2^. This complex ecosystem exists symbiotically with the host and has essential roles in host immunity, metabolism, and nutrition^3^. To foster a healthy gut microbiota balance, regular consumption of beneficial probiotic bacteria is highly recommended. Probiotics are living microorganisms that confer beneficial health effects by modulating the gut microbiota when consumed in sufficient amounts. Probiotic consumption has been shown to have therapeutic and prophylactic effects in a wide array of gastrointestinal diseases and immune disorders associated with gut microbiota dysbiosis^4,5^. Although the health benefits of probiotics have been recognized for centuries, recent interest has shifted toward the optimization of probiotic efficacy. A common strategy to increase the probiotic effect is the inclusion of prebiotics, nondigestible dietary components that benefit the host through promotion of selective bacterial growth in the intestinal tract. Synbiotics, the combination of probiotics and prebiotics, are a promising advancement in the field of functional foods, as conventional probiotics alone have limited implantation and survival in the gastrointestinal tract without the aid of prebiotics^6,7^.

Optimally synergistic probiotic bacterial strains and prebiotics must be selected to develop synbiotic formulations with maximal health benefits. Effective probiotic bacteria are often isolated from fermented foods. Kimchi is a traditional Korean fermented food known to have health-promoting activity because it serves as a rich source of probiotic bacteria. Kimchi contains a variety of lactic acid bacteria (LAB) that have been shown to have antioxidant, immunomodulatory, and antiadipogenic activities^8^. Several species belonging to the genera *Leuconostoc* are LAB commonly found in kimchi and have been previously used as probiotics in a variety of applications, such as silage and prevention of oral biofilm formation^9,10^. *Leuconostoc holzapfelii* (*L*. *holzapfelii*) is one species commonly isolated from kimchi that heterofermentatively produces lactic acid and mannitol, a polyol with beneficial health effects as an antioxidant and nonmetabolizable sugar^11^. However, to our knowledge, the effects of *L*. *holzapfelii* consumption on the gut microbiota composition and blood chemistry of healthy human subjects have yet to be published. Selection of prebiotics with high oligosaccharide content and other beneficial characteristics, such as antioxidant activity, is also critical for optimal synbiotic development. Dietary fibers rich in oligosaccharides are indigestible in the small intestine and pass to the colon, where they are fermented by commensal gut bacteria. Oligosaccharide fermentation has a myriad of beneficial effects, including acidification of the colon, increased populations of *Lactobacilli* and *Bifidobacteria*, increased production of short-chain fatty acids, and increased colon crypt depth^12,13^. Soybeans and broccoli are rich sources of indigestible, fermentable oligosaccharides, making them ideal prebiotics^14^. In addition, food ingredients high in polyphenols and vitamins, such as broccoli, black soybeans, persimmon oil, and fo-ti, have high antioxidant activity, further contributing to their beneficial effect on the gut microbiota^15–18^. Therefore, in this study, a synbiotic beverage was formulated, incorporating kimchi-derived *L*. *holzapfelii* and functional prebiotics high in fermentable carbohydrates and antioxidants, to maximize beneficial gut microbiota effects.

The primary overarching mechanism of probiotic and prebiotic effects on host health is modulation of the gut microbiota. However, it can be difficult to elucidate significant changes in the gut microbiota composition in response to particular probiotic or synbiotic treatments. Therefore, recent efforts have focused on characterizing changes in the functional activities of the microbiome as well as compositional alterations. To decipher the complex, systemic network of interactions between the gut microbiota and human host, recent studies have investigated the role of microbial-derived extracellular vesicles (EVs) in host health^19,20^. Bacterial EVs are ubiquitously released nanovesicles 20–200 nm in diameter that distally transport bacterial proteins, nucleic acids, lipids, and other components to host and bacterial cells throughout the body^21^. In addition to their functional properties in the host, the inclusion of bacterial DNA enables metagenomic analysis of 16S rDNA contained in bacterial EVs, allowing the identification and characterization of bacterial EV activity both locally and systemically.

As the probiotic potential of LAB isolated from fermented foods has become increasingly recognized, it is necessary to understand how the microbiota composition and activity changes in response to the consumption of specific strains and synbiotic formulations. In this study, we conducted a clinical trial in which participants consumed a synbiotic beverage including *L*. *holzapfelii* isolated from kimchi and prebiotics such as black soybean, broccoli, fo-ti, and persimmon vinegar for 4 weeks. Then, 16S rDNA metagenomic analysis was conducted on bacteria isolated from stool as well as microbiota-derived EVs isolated from stool and urine to assess holistic microbiome changes before and after consumption of the *L*. *holzapfelii*-enriched synbiotic beverage. In addition, a wide array of blood chemistry assays were performed to screen for potential blood chemistry alterations induced by the synbiotic intervention.

## Materials and methods

### Subjects

In total, 21 healthy people, 9 males and 12 females, were enrolled from Dankook University Hospital in 2017. Exclusion criteria included being under 20 years old, having cancer or diabetes, being pregnant, taking medication, and participating in another clinical trial. The present study was approved by the Institutional Review Board of Dankook University Hospital (IRB No. DKUH 2017–05–020). The methods conducted in this study were in accordance with the approved guidelines, and informed consent was obtained from all subjects.

### Clinical study design

Ten grams of kimchi was added to 90 ml of sterile distilled water and then shaken for 1 h. After shaking, the solution was serially diluted to a 10^−5^ dilution, plated on solid MRS medium (MB cell, South Korea), and then incubated at 35 °C for 2 days. *L*. *holzapfelii* colonies were isolated by the streak plate method and then lyophilized. Black soybean (*Rhynchosia volubilis*), persimmon vinegar, fo-ti (*Polygonum multiflorum* Thunberg) and broccoli were cultured together at 35 °C for 1 day, and then kimchi-extracted *L. holzapfelii* lyophilisate was added to the resulting beverage. In clinical trials, all volunteers consumed a fixed amount of the *L. holzapfelii-*enriched synbiotic beverage twice daily, in the morning and evening, over the course of 4 weeks. Stool, urine, and blood samples were obtained before and after consumption. Blood samples were processed through a serum separator tube (SST) to separate and collect serum samples. Metagenomic analysis was conducted on EVs isolated from stool and urine samples, and blood chemistry assays were conducted on the separated serum samples.

### EV isolation and DNA extraction

Human stool samples were filtered through a cell strainer after being diluted in 10 ml of PBS for 24 h. To separate EVs from stool and urine samples, EVs in samples were isolated by centrifugation at 10,000 × *g* for 10 min at 4 °C. After centrifugation, the pellet of stool samples contained bacterial cells, and the supernatant of stool and urine samples contained EVs. Bacteria and foreign particles were eliminated from stool and urine sample supernatants by sterilizing the supernatant through a 0.22 µm filter. To extract DNA from bacterial cells and bacterial EVs, bacteria and EVs were boiled for 40 min at 100 °C. To eliminate remaining floating particles and waste, the supernatant was collected after 30 min of centrifugation at 13,000 rpm at 4 °C. DNA was extracted using a DNA isolation kit (PowerSoil DNA Isolation Kit, MO BIO, USA) following the standard protocol in the kit guide. The DNA extracted from the bacterial cells and EVs contained in each sample was quantified using a QIAxpert system (QIAGEN, Germany).

### Bacterial metagenomic analysis of human stool samples

Bacterial genomic DNA was amplified with 16 S_V3_F (5′-TCGTCGGCAGCGTCAGATGTGTATAAGAGACAGCCTACGGGNGGCWGCAG-3′) and 16S_V4_R (5′-GTCTCGTGGGCTCGGAGATGTGTATAAGAGACAGGACTACHVGGGTATCTAATCC-3′) primers specific for V3–V4 hypervariable regions of the 16S rDNA gene. Libraries were prepared using PCR products according to the MiSeq System guide (Illumina, USA) and quantified using QIAxpert (QIAGEN, Germany). Each amplicon was then quantified, set at an equimolar ratio, pooled, and sequenced with MiSeq (Illumina, USA) according to the manufacturer’s recommendations.

### Analysis of the microbiota composition

Raw pyrosequencing reads obtained from the sequencer were filtered according to the barcode and primer sequences using a MiSeq (Illumina, USA). Taxonomic assignment was performed by the profiling program MDx-Pro ver.1 (MD Healthcare, Korea), which selects high-quality sequencing reads with read lengths >300 bp and Phred scores >20 (>99% accuracy of base call). Operational taxonomic units (OTUs) were clustered using the sequence clustering algorithm CD–HIT. Subsequently, taxonomy assignment was carried out using UCLUST and QIIME against the 16S rDNA sequence database in Greengenes 8.15.13. Based on sequence similarities, taxonomic assignment to the genus level was performed on all 16S rDNA sequences. The bacterial composition at each level was plotted in a stacked bar graph. In cases where clusters could not be assigned at the genus level due to lack of sequences or redundant sequences in the database, the taxon was assigned at the next highest level, as indicated in parentheses.

### Blood chemistry

Blood samples were collected in SSTs and centrifuged at 3000 rpm for 15 min at 4 °C to separate the serum from blood samples. Blood chemistry was then assessed by measuring total protein (TP), albumin (ALB), globulin (GLB), glucose (GLU), aspartate aminotransferase (AST), alkaline aminotransferase (ALT), blood urea nitrogen (BUN), total bilirubin (T-BIL), cholesterol (CHO), alkaline phosphatase (ALP), c-reactive protein (CRP), triglycerides (TG), creatine (CREA), gamma glutamyl peptidase (GGT), high-density lipoprotein cholesterol (HDL-C), and low-density lipoprotein cholesterol (LDL-C) levels.

### Statistical analysis

To avoid potential bias caused by differing sequencing depths, samples with >3500 reads were rarefied to a depth of 3500 reads for subsequent analysis. Significant differences before and after consumption of the *L. holzapfelii*-enriched synbiotic beverage were tested using a *t* test for continuous variables. In addition, Pearson correlation analysis was performed between samples. A *p*-value < 0.05 was considered statistically significant. Principal component analysis (PCA) was performed to determine differential clustering of samples before and after consumption of the *L. holzapfelii*-enriched synbiotic beverage. The alpha diversity of microbial composition was measured using the Chao1 index and rarified to compare species richness. Shannon’s index was used to measure the species diversity of samples before and after consumption of the *L. holzapfelii*-enriched synbiotic beverage. All statistical analyses were performed using R version 3.4.1.

## Results

### Blood chemistry

Blood chemistry analysis showed that the average fold-change of AST, TG, and GLB levels before and after ingestion of the synbiotic beverage was 0.63, 0.71, and 0.89, respectively. The fold changes of all other tested blood chemistry analytes were between 0.91 and 1.07. Overall, the most dramatic blood chemistry change observed in the subjects was a 0.18-fold AST reduction. Average serum AST levels were significantly lowered from 34.4 (standard deviation (SD) 22.9) IU/L to 21.7 (SD 8.2) IU/L after consumption of the *L. holzapfelii-*enriched synbiotic beverage (*p* = 0.03) (Table 1). In total, 75% of female participants and 25% of male participants showed decreased serum AST levels, while 50% of the participants showed no noticeable changes in serum AST levels (0.9–1.1-fold change). Subjects with starting AST levels above 40.0 IU/L, between 40.0 IU/L and 20.0 IU/L, and below 20.0 IU/L showed average fold changes of 0.38, 0.99, and 1.13, respectively.

**Table 1:** Blood chemistry before and after synbiotic beverage consumption

Blood chemistry	Reference range^a^	Before mean	SD	After mean	SD	*p-*value
TP [g/dl]	6.6–8.7	7.785	1.487	7.230	0.762	0.1587
ALB [g/dl]	3.5–5.2	4.965	0.901	4.715	0.497	0.2981
GLB [g/dl]	2.3–3.5	2.820	0.741	2.515	0.382	0.1218
A/G ratio		1.820	0.306	1.905	0.248	0.3527
GLU [mg/dl]	<100	103.300	24.220	104.800	18.581	0.8315
AST [IU/L]	0–40	34.400	22.901	21.650	8.242	0.0316*
ALT [IU/L]	0–40	8.600	3.555	9.100	4.625	0.7108
BUN [mg/dl]	10–25	12.945	3.809	11.920	3.265	0.3788
T-BIL [mg/dl]	0.1–1.2	0.606	0.204	0.550	0.200	0.3986
CHO [mg/dl]	Ideal: 200 Borderline: 201–239 High: ≥240	205.300	49.455	202.850	35.702	0.8619
ALP [U/L]	40–120	36.400	13.811	36.650	9.850	0.9491
CRP [mg/dl]	0.00–0.49	0.060	0.058	0.064	0.064	0.8412
TG [mg/dl]	Ideal: 0–150 Borderline: 150–199 High: 200–499 Very high: ≥500	176.200	205.642	124.400	87.692	0.3219
CREA [mg/dl]	0.7–1.4	0.996	0.317	0.910	0.175	0.3056
GGT [U/L]	Male: 10–71 Female: 6–42	30.600	30.681	29.600	35.311	0.9262
HDL-C [mg/dl]	Male: ≥40 Female: ≥50	64.200	15.763	60.250	12.397	0.3960
LDL-C [mg/dl]	0–100	122.400	33.131	121.050	28.273	0.8932

*TP* total protein, *ALB* albumin, *GLB* globulin, *GLU* glucose, *AST* aspartate aminotransferase, *ALT* alkaline aminotransferase, *BUN* blood urea nitrogen, *T-BIL* total bilirubin, *CHO* cholesterol, *ALP* alkaline phosphatase, *CRP* c-reactive protein, *TG* triglycerides, *CREA* creatine, *GGT* gamma glutamyl peptidase, *HDL-C* high-density lipoprotein cholesterol, *LDL-C* low-density lipoprotein cholesterol

^a^Reference range according to Asan Medical Center, Korea

*p < 0.05

### Microbiome analysis of stool bacteria

**Fig. 1:**
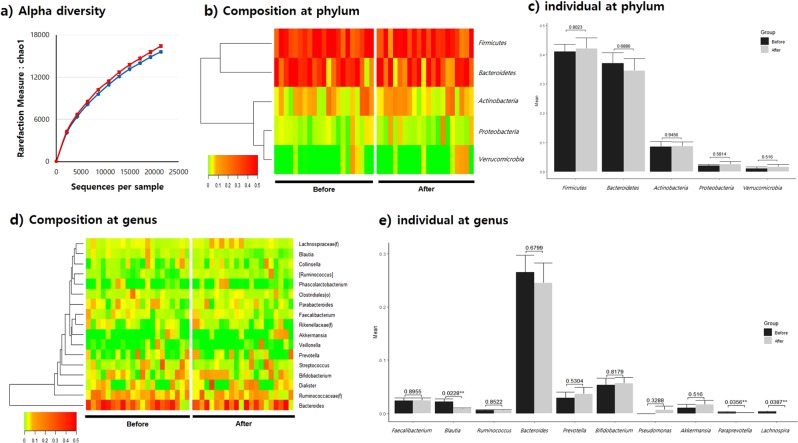
Composition of stool bacteria before and after consumption of a synbiotic beverage. **a** Alpha diversity measured by Chao1, **b** composition of the microbiome at the phylum level, **c** individual microbiome at the phylum level, **d** composition of the microbiome at the genus level, and **e** individual microbiome at the genus level

Chao1 and Shannon index alpha diversity analysis did not show any obvious changes in species richness before and after *L. holzapfelii-*enriched synbiotic beverage consumption (Fig. 1a, Supplementary Fig. 1a). The average number of OTUs before and after consumption was slightly decreased from 118.0 to 112.0. Stool bacterial samples had an average coverage of 41,384.8 (SD 18,414.3) and 37,133.1 (SD 18,922.4) valid reads per sample before and after consumption, respectively. PCA of stool bacteria showed no significant differences before and after ingestion (Supplementary Fig. 2a, d). Analysis of microbiome abundances at the phylum level revealed that Firmicutes, Bacteroidetes, Actinobacteria, and Proteobacteria were the most abundant phyla, in that order (Fig. 1b, c). There were no statistically significant differences before and after consumption in any taxa at the phylum level. At the genus level, *Bacteroides*, followed by *Ruminococcaceae (f)*, was the most abundant taxa, and *Blautia*, *Lachnospira*, and *Paraprevotella* abundances were significantly decreased after ingestion (*p* < 0.05) (Fig. 1d, e). *Pseudomonas*, *Streptophyta (o)*, and *Acinetobacter* abundances were sharply increased by 521.84-, 16.82-, and 12.13-fold, respectively; however, there was no statistical significance.

### Microbiome analysis of stool EVs

**Fig. 2:**
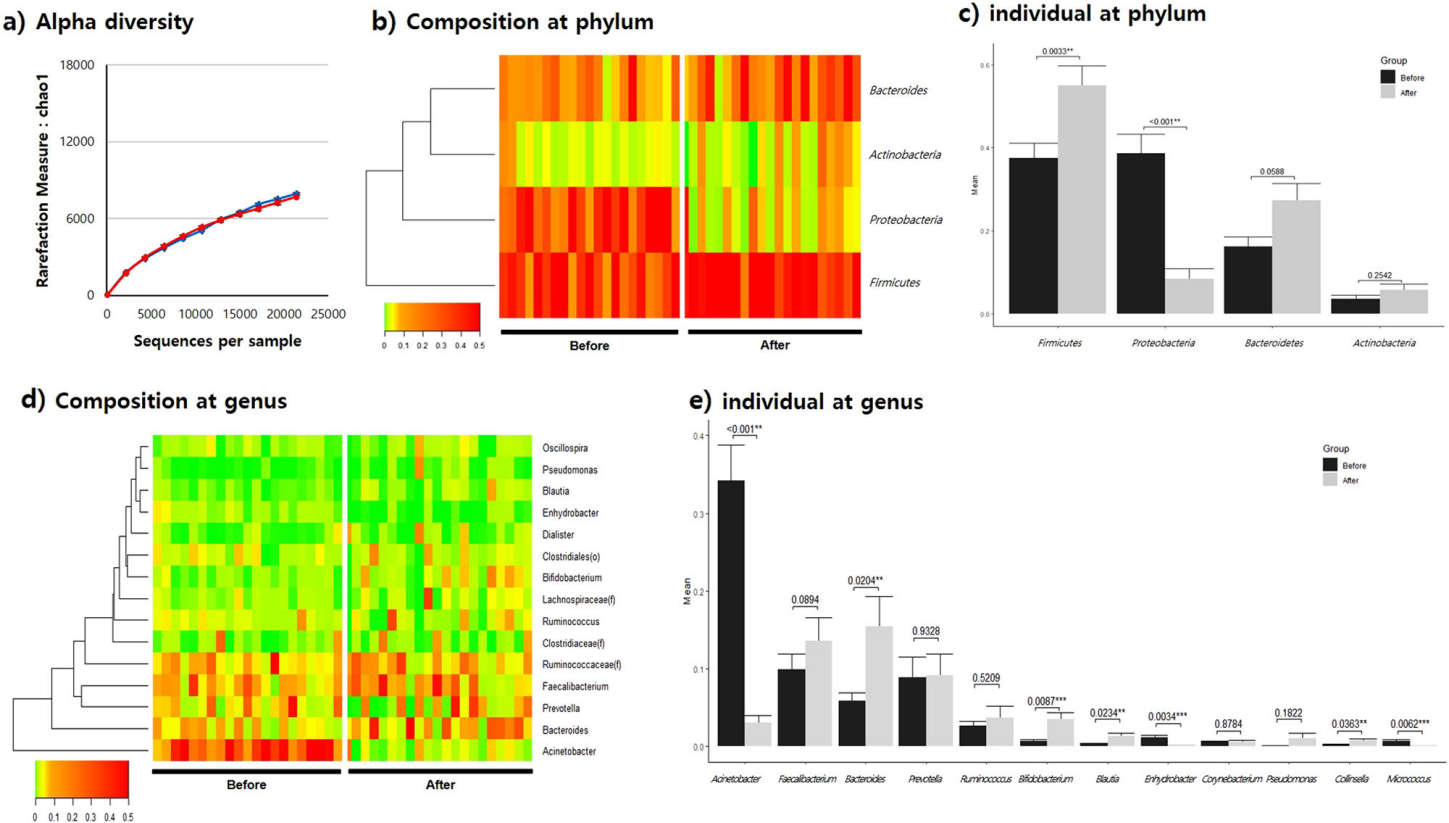
Composition of microbiota-derived stool EVs before and after consumption of a synbiotic beverage. **a** Alpha diversity measured by Chao1, **b** composition of the microbiome at the phylum level, **c** individual the microbiome at the phylum level, **d** composition of the microbiome at the genus level, and **e** individual microbiome at the genus level

Species richness before and after *L. holzapfelii-*enriched synbiotic beverage consumption was shown to be comparable in stool bacterial EVs through Chao1 and Shannon index alpha diversity analysis (Fig. 2a, Supplementary Fig. 1b**)**. The average number of OTUs from before to after consumption was moderately increased from 86.8 to 93.6, respectively. Furthermore, the average valid reads of stool bacterial EVs showed an increased tendency, from 38,928.5 (SD 18,465.5) to 40,435.8 (SD 20,577.2) valid reads from before to after consumption, respectively. PCA analysis of stool EVs showed a significant difference at the phylum and genus levels before and after ingestion of the synbiotic beverage (Supplementary Fig. 2b, e). At the phylum level, Firmicutes, Proteobacteria, Bacteroides, and Actinobacteria were the most predominant taxa. After consumption, Firmicutes abundance was significantly increased and Proteobacteria abundance significantly decreased (*p* < 0.01), with fold-changes of 1.47 and 0.22, respectively (Fig. 2b, c). At the genus level, *Acinetobacter*, *Enhydrobacter*, and *Micrococcus* abundances were significantly decreased, and *Bacteroides*, *Bifidobacterium*, and *Blautia* abundances significantly increased (*p* < 0.05) after consumption of the synbiotic beverage. *Acinetobacter* and *Bifidobacterium* abundances showed particularly significant fold changes of 0.09 and 5.12, respectively (Fig. 2d, e). *Blautia* stool EVs were shown to increase inversely with those of stool bacteria. Finally, although not statistically significant, *Pseudomonas* stool EVs showed a fold change of 7.87, which is in line with the increase in *Pseudomonas* bacteria measured in stool.

### Microbiome analysis of urinary EVs

**Fig. 3:**
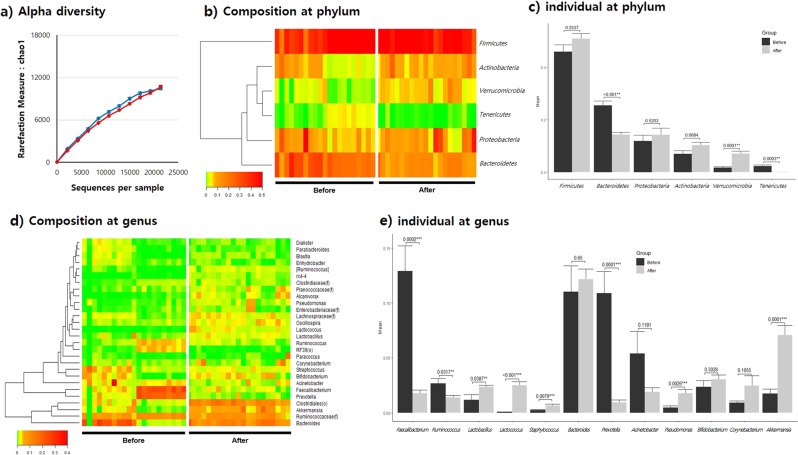
Composition of microbiota-derived urinary EVs before and after consumption of a synbiotic beverage. **a** Alpha diversity measured by Chao1, **b** composition of the microbiome at the phylum level, **c** individual microbiome at the phylum level, **d** composition of the microbiome at the genus level, and **e** individual microbiome at the genus level

Chao1 alpha diversity analysis did not show any obvious changes in species richness; however, a significant change (*p* < 0.001) in Shannon index diversity was observed before and after synbiotic beverage consumption (Fig. 3a, Supplementary Fig. 1c). Furthermore, the number of OTUs before and after *L. holzapfelii-*enriched synbiotic beverage consumption slightly decreased from 112.4 to 110.5. The average valid reads of urine microbial EVs increased from 19,267.4 (SD 12,819.5) to 24,372.2 (SD 11,787.1) from before to after consumption, respectively. PCA analysis revealed that while there was a significant difference between groups at the phylum level, no distinct clustering was observed at the genus level before and after consumption of the *L. holzapfelii*-enriched synbiotic beverage (Supplementary Fig. 2c, f). At the phylum level, Firmicutes accounted for the largest proportion of urinary EVs, at 46.0% before and 50.8% after consumption, followed by Bacteroidetes, Proteobacteria, and Actinobacteria. Bacteroidetes and Tenericutes abundances were significantly decreased, and Verrucomicrobia abundance significantly increased (*p* < 0.01), with Tenericutes and Verrucomicrobia abundances exhibiting fold-changes of 0.07 and 4.06, respectively (Fig. 3b, c). At the genus level, *Faecalibacterium*, *Ruminococcus*, and *Prevotella* abundances were significantly decreased after consumption. Conversely, after synbiotic beverage consumption, *Lactobacillus*, *Lactococcus*, *Staphylococcus*, *Pseudomonas*, and *Akkermansia* abundances were significantly increased (Fig. 3d, e).

### Correlation of samples

Correlation analysis of the overall microbiome distribution between patient samples before and after synbiotic beverage consumption revealed that all patients were highly correlated at the phylum level (Table 2a). However, at the genus level, stool bacteria before ingestion and after ingestion showed the strongest correlation of 0.72 (SD 0.24). There was a significant correlation between stool bacteria and urinary EVs before consumption. The correlation between stool bacteria and stool EVs was 0.56 (SD 0.26), and the correlation between stool bacteria and urinary EVs was 0.50 (SD 0.17) after consumption. Stool bacteria and urinary EVs before ingestion were significantly correlated with all samples after ingestion, and stool EVs after consumption were highly correlated with all samples before ingestion (Table 2b).

**Table 2:** Correlation between the microbiota composition of sample types before and after synbiotic beverage consumption at the a) phylum and b) genus levels

	Be-S-B	Be-S-E	Be-U-E	Af-S-B	Af-S-E	Af-U-E
(a) Phylum level sample correlation						
Be-S-B	1.0000					
Be-S-E	0.5769	1.0000				
Be-U-E	0.8327	0.6896	1.0000			
Af-S-B	0.8202	0.5621	0.8201	1.0000		
Af-S-E	0.8053	0.6450	0.8216	0.8437	1.0000	
Af-U-E	0.7526	0.7440	0.8407	0.7441	0.8155	1.0000
(b) Genus level sample correlation						
Be-S-B	1.0000					
Be-S-E	0.3022	1.0000				
Be-U-E	0.4941	0.3846	1.0000			
Af-S-B	0.7186	0.2889	0.4346	1.0000		
Af-S-E	0.4802	0.4555	0.4047	0.5560	1.0000	
Af-U-E	0.5433	0.3011	0.4363	0.4952	0.3947	1.0000

*Be* before, *Af* after, *S-B* stool bacteria *S-E* stool EVs, *U-E* urinary EVs

As a result of correlation analysis between individual strains, the correlation coefficient between Verrucomicrobia and Firmicutes abundances was 0.55 and 0.33 between stool bacteria and stool EVs, respectively, at the phylum level. Bacteroidetes and Proteobacteria abundances showed a negative correlation between stool EVs and urinary EVs; however, Proteobacteria abundance was not significantly correlated (Table 3a). At the genus level, *Bacteroides*, *Ruminococcaceae (f)*, *Prevotella*, *Planococcaceae (f)*, and *Akkermansia* abundances showed a strong correlation between stool bacteria and stool EVs. *Parabacteroides* and *Blautia* abundances showed a weak negative correlation, although there was no statistical significance. *Bacteroides* and *Lachnospiraceae (f)* abundances showed a positive correlation between stool bacteria and urinary EVs. *Faecalibacterium* and *Lactococcu*s abundances showed a negative correlation; however, this correlation was not significant. In addition, *Streptococcus*, *Lactococcus*, *RF39 (o)*, and *Alcanivorax* abundances showed a significant positive correlation between stool EVs and urinary EVs (Table 3b).

**Table 3:** Correlation between individual taxa of each sample type

Taxon	Stool-Bac		Stool-EV		Urine-EV		Correlation		
	Mean	SD	Mean	SD	Mean	SD	S-B/S-E	S-B/U-E	S-E/U-E
(a) Phylum level taxon correlation									
Firmicutes	0.416	0.143	0.463	0.210	0.484	0.109	0.327*	0.037	0.044
Bacteroidetes	0.359	0.177	0.218	0.160	0.199	0.082	0.205	0.201	−0.319*
Proteobacteria	0.023	0.031	0.235	0.226	0.130	0.110	−0.136	−0.009	−0.233
Actinobacteria	0.087	0.073	0.047	0.054	0.087	0.055	0.196	−0.099	0.282
Verrucomicrobia	0.014	0.033	0.003	0.005	0.044	0.041	0.546**	−0.068	0.109
Tenericutes	0.000	0.001	0.002	0.003	0.012	0.019	0.310*	−0.022	0.472**
(b) Genus level taxon correlation									
*Bacteroides*	0.256	0.158	0.107	0.141	0.116	0.083	0.511**	0.329*	0.205
*Ruminococcaceae(f)*	0.095	0.066	0.097	0.101	0.085	0.029	0.426**	−0.068	−0.089
*Faecalibacterium*	0.025	0.022	0.118	0.117	0.074	0.094	0.063	−0.263	−0.234
*Clostridiales(o)*	0.024	0.034	0.024	0.031	0.071	0.055	0.196	0.038	0.014
*Prevotella*	0.033	0.054	0.091	0.121	0.059	0.082	0.544**	−0.091	0.005
*Bifidobacterium*	0.055	0.056	0.021	0.033	0.027	0.023	0.363*	−0.011	0.204
*Streptococcus*	0.040	0.069	0.005	0.006	0.030	0.031	−0.008	−0.138	0.381*
*Dialister*	0.054	0.067	0.014	0.032	0.008	0.009	0.182	−0.072	−0.121
*Akkermansia*	0.014	0.033	0.003	0.004	0.044	0.041	0.554**	−0.068	0.116
*Lachnospiraceae(f)*	0.032	0.023	0.021	0.052	0.021	0.013	0.060	0.315*	0.121
*Parabacteroides*	0.033	0.032	0.005	0.008	0.007	0.009	−0.201	−0.037	−0.082
*Acinetobacter*	0.001	0.008	0.186	0.218	0.037	0.069	−0.103	−0.049	0.008
*Oscillospira*	0.009	0.009	0.011	0.014	0.020	0.014	0.035	0.136	−0.082
*Collinsella*	0.021	0.033	0.005	0.008	0.006	0.006	−0.027	0.019	0.183
*Clostridiaceae(f)*	0.007	0.013	0.022	0.053	0.010	0.006	0.045	0.045	0.047
*Rikenellaceae(f)*	0.022	0.029	0.006	0.018	0.005	0.007	0.049	0.013	−0.139
*Ruminococcus*	0.006	0.006	0.032	0.054	0.020	0.017	0.293	0.092	−0.031
*Blautia*	0.016	0.019	0.009	0.014	0.009	0.008	−0.171	−0.162	0.042
*[Ruminococcus]*	0.013	0.025	0.002	0.004	0.010	0.009	0.308*	0.207	−0.033
*Lactobacillus*	0.001	0.004	0.003	0.010	0.018	0.018	−0.066	−0.030	0.033
*Corynebacterium*	0.000	0.000	0.006	0.008	0.017	0.030	0.109	−0.026	−0.049
*Pseudomonas*	0.004	0.023	0.006	0.022	0.011	0.013	−0.040	−0.109	0.121
*Phascolarctobacterium*	0.012	0.026	0.003	0.013	0.003	0.005	−0.054	0.048	0.046
*Planococcaceae(f)*	0.001	0.003	0.005	0.007	0.013	0.015	0.829**	0.227	0.190
*Lactococcus*	0.000	0.001	0.000	0.000	0.013	0.016	−0.147	−0.226	0.324*
*RF39(o)*	0.000	0.001	0.002	0.003	0.012	0.019	0.312*	−0.020	0.472**
*Alcanivorax*	0.000	0.001	0.001	0.002	0.012	0.016	0.197	0.139	0.333*

*Be* before, *Af* after, *S-B* stool bacteria, *S-E* stool EVs, *U-E* urinary EVs

*p < 0.05, **p < 0.01

## Discussion

In this study, we identified significant alterations in the gut microbiota composition and blood chemistry after consumption of an *L. holzapfelii-*enriched synbiotic beverage. This report is the first study to characterize significant changes in microbiota-derived stool and urinary EVs following synbiotic treatment, providing a novel tool for microbiome analysis in clinical studies. Our results demonstrated that prolonged consumption of a *L. holzapfelii-*enriched synbiotic beverage decreased average serum AST levels. Elevated levels of AST in serum are associated with poor liver function and are predictors of diseases such as liver cancer, non-alcoholic fatty liver disease (NAFLD), cirrhosis, and overall mortality from liver disease^22–24^. Clinicians typically regard 40.0 IU/L as the upper-limit of the normal reference for AST serum levels^25^. In this clinical trial, subjects with starting serum AST levels over 40.0 IU/L exhibited much greater fold-changes in AST levels after *L. holzapfelii-*enriched synbiotic beverage consumption than those with starting levels below 40.0 IU/L. These results are consistent with reports of decreased serum aminotransferase levels in NAFLD patients after treatment with probiotic LAB as standalone as well as synbiotic formulations^26–28^.

A variety of mechanisms through which probiotic LAB improve liver function have been proposed, including inflammatory response inhibition and reduction of oxidative damage, TNFα levels, and apoptosis^29–32^. Another possible mechanism by which the *L. holzapfelii*-enriched synbiotic beverage used in this study may have decreased AST levels is the production of folate, a B vitamin critical for cell function and metabolism^33^. Folate deficiency has been associated with increased AST levels, and folic acid supplementation has been shown to decrease aminotransferase levels in previous clinical studies^34,35^. Broccoli and soybeans are also known to contain substantial amounts of folate, which may further enhance the beneficial liver effect of the synbiotic beverage consumed in this study^36^. Altogether, these findings indicate that *L. holzapfelii-*enriched synbiotic beverage consumption may confer protection against liver disease through reduction of hepatic damage, particularly in those with abnormally high starting AST serum levels. As all enrolled participants in this study were healthy volunteers, further study in patients with liver dysfunction should be conducted to verify this beneficial effect, particularly the potential impact of LAB-associated BMI reduction in patients with fatty liver disease.

This clinical trial is also the first of its kind to combine microbiota-derived stool and urinary EV metagenomic analysis with conventional stool bacteria composition into a novel microbiome analysis method. The utility of this method is apparent, as the majority of the statistically significant microbiome alterations assessed in this study were observed in stool and urinary EVs rather than in stool bacteria. Through this novel method, we were able to determine a variety of significant changes in the proportions of bacterial EVs released locally in stool and systemically in urine despite limited gut bacterial microbiota alterations. Several of the altered taxa, such as *Akkermansia*, *Bacteroides*, *Prevotella*, Lachnospiraceae, and Proteobacteria, have previously been shown to have clinical relevance. Proteobacteria are commonly in low abundance in healthy guts, while conversely, Proteobacteria blooms are associated with disease states and gut dysbiosis. A high abundance of Proteobacteria can lead to dysbiosis states that are associated with metabolic disorders, inflammation, and cancer^37^. Furthermore, increased Proteobacteria EV abundance was detected in the stool of mice with high-fat-diet-induced insulin resistance associated with type 2 diabetes and other metabolic conditions^38^. High levels of *Prevotella* are also associated with chronic inflammation, which can promote a variety of different diseases^39^. Therefore, the decreased *Prevotella* and Proteobacteria EV abundances induced by *L. holzapfelii* synbiotic consumption may contribute to a healthier microbiome balance through reduction of inflammatory taxon activity.

Conversely, several taxa that were significantly increased after *L. holzapfelii* consumption are known to protect against overweight and obese phenotypes. The *Akkermansia* genus shown to increase in abundance in this study consists of primarily *Akkermansia muciniphila*, a beneficial bacterial species known to be inversely associated with obesity, inflammation, and other metabolic diseases^40,41^. We demonstrated in a previous study that *A. muciniphila* EVs were decreased in an experimental irritable bowel disease (IBD) mouse model. Further, it was revealed in this study that treatment with *A. muciniphila* EVs was able to prevent IBD phenotypes in the IBD model^19^. Meanwhile, *Bacteroides* spp. in the gut generally retain a healthy symbiotic relationship with the host, and in this study, a significant increase in stool EVs was observed, while a slight decreasing trend was observed in urinary EVs^42^. A previous dietary intervention study found that *Bacteroides* abundance was increased in obese adolescents upon significant weight loss. The authors suggested that a *Bacteroides* bloom could increase propionate levels, leading to lipid synthesis inhibition and lean phenotype promotion^43^. Increased abundance of Lachnospiraceae, a common family in the Firmicutes phylum comprised of butyrate-producing bacteria, has also been reported in other probiotic studies administering another kimchi-derived LAB probiotic, *Lactobacillus plantarum*. These increases in Lachnospiraceae abundance have been associated with decreased body weight as well as upregulated expression of lipid oxidative genes in mice^44,45^. In light of these findings, it is possible that prolonged consumption of the *L. holzapfelii*-enriched synbiotic beverage could contribute to lean phenotype promotion by increasing *Akkermansia*, *Bacteroides*, and Lachnospiraceae EV activity.

EVs released by bacteria in the gut can transverse the intestinal barrier and enter circulation, functioning in distal intercellular communication between the microbiota and host^20^. Microbiota-derived EVs are known to have roles in immunomodulation, horizontal gene transfer, and metabolism, making them potent targets for assessing both local and systemic microbiota activity^38,46,47^. EVs circulating systemically in the body and released in urine showed particularly significant alterations in Shannon species diversity, PCA, and individual taxon abundance at both the phylum and genus levels. These results demonstrate that while bacterial composition itself was marginally impacted by *L. holzapfelii*-enriched synbiotic beverage consumption, microbiota activity via EV biogenesis and release was greatly altered. This finding is significant, as it highlights the need for a wider analysis of the microbiota composition and activity to accurately assess holistic microbiome alterations in future clinical dietary intervention studies. Furthermore, the health effects of the significant changes in stool and urine EVs caused by *L. holzapfelii*-enriched synbiotic consumption should be further verified in future studies targeting patients in varying disease states.

The correlation between the total microbiota composition as well as individual taxon abundance was also assessed between stool bacterial, stool EV, and urinary EV samples before and after synbiotic beverage consumption. Although stool bacteria composition was highly correlated before and after synbiotic beverage consumption at both the phylum and genus levels, stool EV and urinary EV samples showed considerably less correlation than stool bacterial samples before and after consumption, particularly at the genus level. Furthermore, individual taxa were the most strongly correlated between stool bacteria and stool EV samples, particularly the Verrucomicrobia phylum, Planococcaceae family, and *Bacteroides*, *Prevotella*, and *Akkermansia* genera. Conversely, there was a very little correlation between stool EVs and urinary EVs, highlighting the unique composition of microbiota-derived EVs released locally in stool and circulating systemically in urine.

An interesting finding from the correlation analysis of individual taxon abundance between sample groups was that while certain taxa such as *Bacteroides*, *Ruminococcaceae (f), Prevotella*, and *Akkermansia* were positively correlated, other taxa such as *Ruminococcus* were negatively correlated between stool bacteria and stool EVs. This disparity in correlation directionality is likely due to the dual mechanism of bacterial EV release. As bacterial cells proliferate, both gram-positive and gram-negative bacteria secrete EVs, outer membrane vesicles (OMVs) and membrane vesicles (MVs), respectively^19^. As bacterial populations grow, this growth in turn causes elevated levels of secreted EVs, explaining the positive correlation observed between particular taxa in stool bacteria and stool EVs. However, there were also taxa such as *Ruminococcus* that showed a negative correlation between stool bacteria and stool EVs. This phenomenon can be explained by another less-recognized EV biogenesis method in bacteria: apoptosis. When bacteria undergo apoptosis, they release apoptotic bodies that deliver bacterial components to cells throughout the body in a method similar to that of bacterial OMVs and MVs^48,49^. Therefore, the negative correlation between *Ruminococcus* stool bacteria and EVs was likely the result of release of apoptotic bodies caused by selective death of *Ruminococcus* bacteria in the gut in response to *L. holzapfelii* synbiotic beverage consumption.

In this study, we were able to associate compositional changes in the intestinal microbiota and microbiota-derived EVs in stool and urine at the genus level with decreased serum AST levels in healthy participants after prolonged consumption of a *L. holzapfelii-*enriched synbiotic beverage. Furthermore, here, we leveraged for the first time microbiota-derived EV metagenomic analysis of stool and urine to characterize complex microbiome alterations induced by synbiotic beverage consumption that were undetectable through conventional bacterial metagenomic analysis. This method can be used in future clinical studies to further characterize microbiome dynamics in synbiotic formulations tailored to different disease states. One such possible application is the use of microbiota-derived EVs as surrogate markers in future clinical trials focused on specific disease states, such as NAFLD.

## Supplementary information

Supplementary Materials.
