# Supplementary material for: Consumption of a Leuconostoc holzapfelii-enriched synbiotic beverage alters the composition of the microbiota and microbial extracellular vesicles
Source: Exp Mol Med. 2019 Aug 1;51(8):87. doi: 10.1038/s12276-019-0288-1 (PMC6802649; doi:10.1038/s12276-019-0288-1)

Supporting information

Supplementary Figure 1. Shannon index before and after consumption of synbiotic beverage

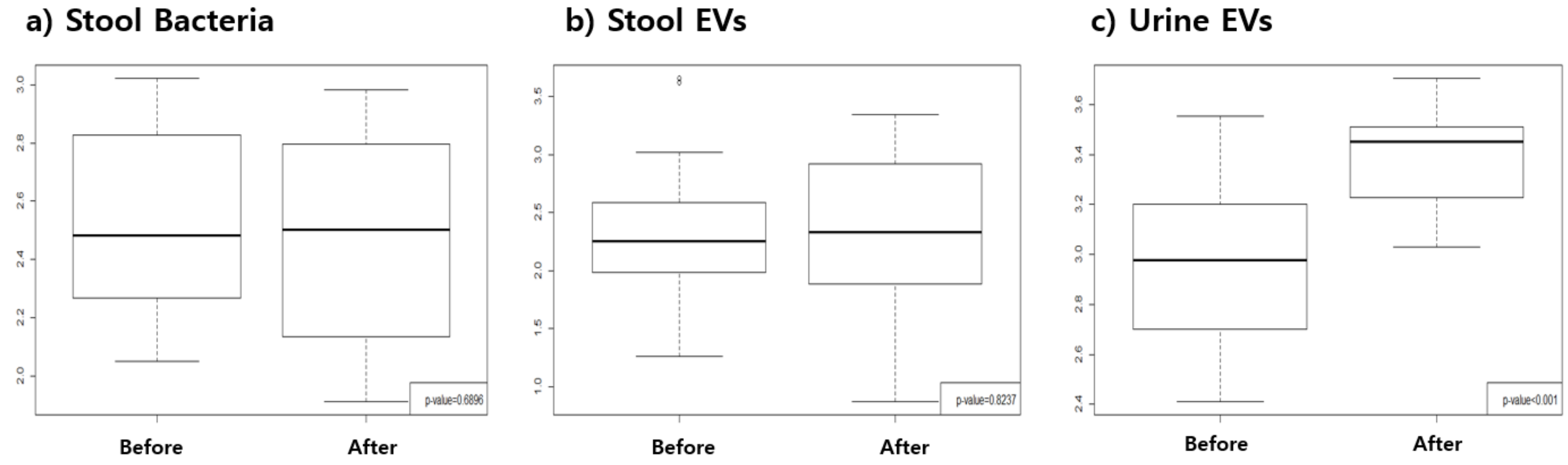

Supplementary Figure 2. PCA (Principle Component Analysis) before and after consumption of symbiotic beverage

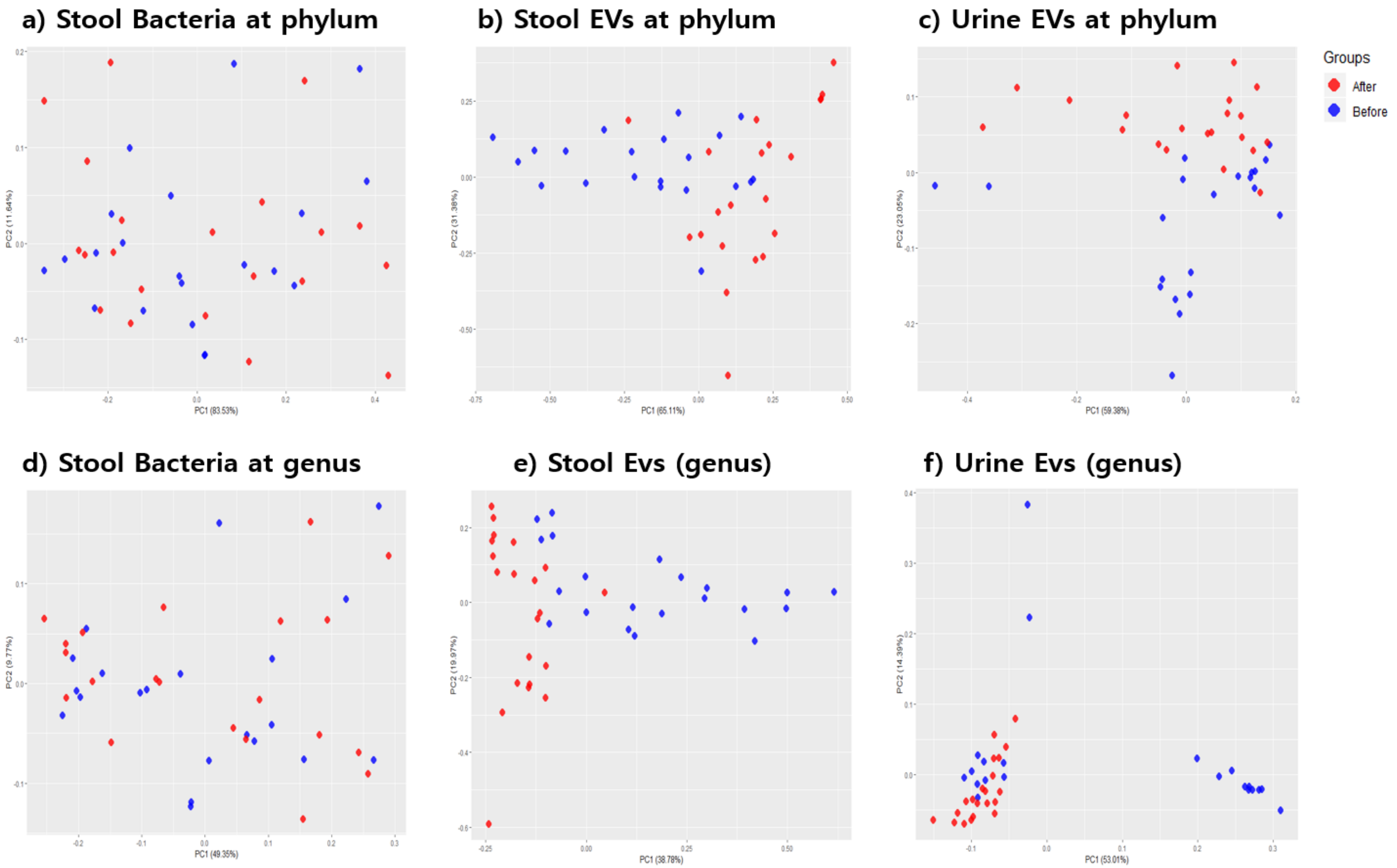

Supplement: Supplementary file 1 — Supplementary Materials. [file 12276_2019_288_MOESM1_ESM.pdf]
